# SG-APSIC1097: The impact of COVID-19 on the incidence of carbapenem-resistant Enterobacterales (CRE) in Singapore: An interrupted time-series analysis

**DOI:** 10.1017/ash.2023.72

**Published:** 2023-03-16

**Authors:** Chong Hui Clara Ong, Thoon Koh Cheng, Surinder Kaur M S Pada, Deepak Rama Narayana, Say Tat Ooi, Nares Smitasin, Kok Choon Raymond Fong, Tse Hsien Koh, Pei Zhi Benjamin Cherng, Raymond Lin, De Partha Pratim, Hsu Li Yang, Jeanette Teo, Oon Tek Ng, Indumathi Venkatachalam, Kalisvar Marimuthu

**Affiliations:** National Centre for Infectious Diseases, Singapore; Paediatrics Infectious Diseases Service, K K Women’s and Children’s Hospital, Singapore; Medicine, Infectious Diseases, Ng Teng Fong General Hospital, Singapore; Laboratory Medicine, Khoo Teck Puat Hospital, Singapore; General Medicine, Khoo Teck Puat Hospital, Singapore; Medicine, National University Hospital, Singapore; Infectious Diseases, Changi General Hospital, Singapore; Microbiology, Singapore General Hospital, Singapore; Infectious Diseases, Singapore General Hospital, Singapore; National Public Health Laboratory, National Centre for Infectious Diseases, Singapore; Laboratory Medicine, Tan Tock Seng Hospital, Singapore; Saw Swee Hock School of Public Health, National University of Singapore, Singapore; CSS Laboratory Medicine, National University Hospital, Singapore; Infectious Diseases, Tan Tock Seng Hospital, Singapore; Infection Prevention & Epidemiology and Infectious Diseases, Singapore General Hospital, Singapore; Infectious Diseases, Tan Tock Seng Hospital, Singapore

## Abstract

**Objectives:** Over the past 2 years, many infection prevention and control (IPC) resources have been diverted to manage the COVID-19 pandemic. Its impact on the incidence of antimicrobial-resistant organisms has not been adequately studied. We investigated the impact of the pandemic on the incidence of carbapenem-resistant Enterobacterales (CRE) in Singapore. **Methods:** We extracted data on unique CRE isolates (clinical and/or surveillance cultures) and patient days for 6 public hospitals in Singapore from the carbapenemase-producing Enterobacteriaceae (CaPES) study group database, and we calculated the monthly incidence of CRE (per 10,000 patient days). Interrupted time-series (ITS) analysis was conducted with the pre–COVID-19 period defined as before February 2020, and the COVID-19 period defined as after February 2020. Statistical analyses were performed using Stata version 15 software. **Results:** From January 2017 to March 2021, 6,770 CRE isolates and 9,126,704 patient days were documented. The trend in CRE monthly incidence increased significantly during the pre–COVID-19 period (0.060; 95% CI, 0.033–0.094; *P* < .001) but decreased during the COVID-19 period (−0.183; 95% CI, −0.390 to 0.023; *P* = .080) without stepwise change in the incidence (−1.496; 95% CI, −3.477 to 0.485; *P* = .135). The trend in monthly incidence rate of CRE clinical cultures increased significantly during the pre–COVID-19 period (0.046; 95% CI, 0.028–0.064; *P* < .001) and decreased significantly during COVID-19 period (−0.148; 95% CI, −0.249 to −0.048; *P* = .048) with no stepwise change in the incidence (−0.063; 95% CI, −0.803 to 0.677; *P* = .864). The trend in monthly incidence rate of CRE surveillance cultures decreased during the pre–COVID-19 period (−0.020; 95% CI, −0.062 to 0.022; *P* = .341) and the COVID-19 period (−0.067; 95% CI, −0.291to 0.158; *P* = .552) without stepwise change in the incidence (−1.327; 95% CI, −3.535 to 0.881; *P* = .233). **Conclusions:** The rate of CRE in clinical cultures decreased during COVID-19 but not the rate in surveillance cultures. Further studies are warranted to study the impact of COVID-19 on CREs.

(DUPLICATE DELETED)

